# Women in chemistry: Q&A with Professor Aurora J. Cruz-Cabeza

**DOI:** 10.1038/s42004-025-01432-2

**Published:** 2025-02-07

## Abstract

Dr Aurora J. Cruz-Cabeza is a Professor of Materials Chemistry at Durham University, in the field of molecular crystals and crystallisation.

**Figure:**
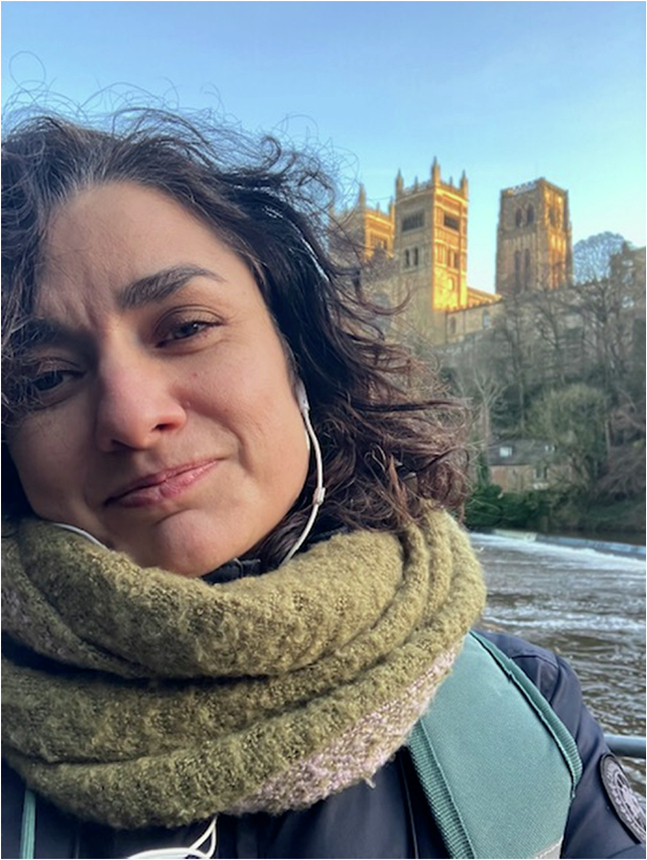
Aurora J. Cruz-Cabeza

Aurora J. Cruz-Cabeza was born in Jaén, one of the most beautiful cities in Spain. She was fascinated by the world from an early age with a particular love for stars and music. She bought her first guitar at the age of 11 and her first telescope at the age of 15. Aurora wanted to study astrophysics but was unable to do so due to geographical constraints, so she decided to move her passion for the big to a passion for the small and studied chemistry instead. She completed two BSc degrees simultaneously, in Chemistry and in Music with classical guitar as her lead instrument. After earning a master’s degree in catalysis in Cordoba (Spain), she moved to the University of Cambridge (UK) where she earned a PhD in Physical Chemistry. At Cambridge, she fell in love with molecular crystals and learned all sorts of techniques to study them from modelling to diffraction. Aurora was the guitarist and second lead voice in a band called “Los Elementos”; they played in numerous gigs around Cambridge through the years.

After PhD, Aurora took several scientific jobs in the UK, the Netherlands and Switzerland. In 2015, Aurora moved to the University of Manchester (UK) where she started her independent academic career as a Lecturer in the Department of Chemical Engineering. At Manchester, she had the huge privilege to work alongside Prof Roger Davey who really influenced her academically. In 2022, she moved to Durham (UK) as a Professor of Materials Chemistry. Aurora has won numerous prizes including the 2023 BCA Prize Lecture and published nearly 100 scientific papers. She is indebted to the people she has had the pleasure to work with during her career, from key mentors and colleagues to students and postdocs whose enthusiasm and hard work always made her group a special one to be part of.

Why did you choose to be a scientist?

I have always been very curious about the world and wanted to learn how things work. From an early age my parents noticed how “I was always inventing things”. At school I was taught science and completely fell in love with mathematics, physics and chemistry. As a teen I was especially taken by the stars since they are scientifically fascinating but also incredibly beautiful. Through the years I ended up working in the field of molecular crystals and crystallization. Like the stars, crystals are beautiful and fascinating. I have now been working with crystals for over 20 years, I am the first person in my family to become a scientist and certainly the first to obtain a master’s degree, a PhD and become a professor. A nurturing school, access to education in various excellent universities around Europe and academic support from lots of people allowed me to pursue a career in science. I am privileged and grateful to have achieved that, though it has certainly required high levels of commitment and dedication.

What scientific development are you currently most excited about?

I am most excited about the generation and use of data in our field. I believe these data will help us push the boundaries of what we know and what we can do with molecular crystals. Our field is quite unique in that thanks to the Cambridge Structural Database (CSD) we are now approaching one and a half million experimental crystal structures available to learn from. Through the years, I have done much of my research using the CSD, where sophisticated data analysis has helped me ask many complex questions about crystals. With new data from electron diffraction, computational data and other physical data for crystals which is only now starting to be collected and archived, I believe we will be able to gather significant learnings from a variety of data in the coming decade. I am excited about this, and we will need to find the right questions for the right data.

What direction do you think your research field should go in?

We need to move from ‘static and structural studies of crystals’, to a field that pursues ‘crystals in action’. And by this I mean, understanding the dynamics of crystals, understanding the birth of crystals (nucleation), their growth and their properties. I am especially eager to see more experimental and computational techniques inform each other to help us understand the complexity of crystal nucleation.

What aspects of your research do you find most exciting or most rewarding?

All careers, scientific or not, go through phases. As a young scientist one is mostly interested in the science, in learning and in pushing the boundaries of our scientific knowledge. As I have become more senior, whilst the scientific curiosity is always a drive, I have found working with and developing people to be *the* most rewarding aspect of my job. I can certainly say that helping members of my group and younger colleagues develop has been a huge privilege for me. You build an academic family that is always there no matter what. With that in place, excellent science thrives.

What impact has your gender had on your career as a scientist?

It is difficult to separate or ascribe career path challenges or opportunities to gender and other personal characteristics. There is, however, significant proven gender bias in academia and we must all recognise this and actively challenge it. All aspects of the academic job suffer from gender bias: from teaching evaluations to reviews of scientific articles and grants to administrative duties assignments and load. In that regard, I am sure my career has suffered from such biases, though they are indeed difficult to quantify. What I can certainly quantify is the support I have had from some important mentors and colleagues (men and women). I feel extremely grateful to many people who have supported me through the years.

Have you been a minority as a woman at any stage of your career? What was that experience like for you?

I have always been a minority as a woman in Chemistry, especially after PhD, and although I have supervised many women, I have only worked under men. Most of them have always been supportive and incredibly encouraging so I have felt very comfortable. I also left my country of origin to pursue a PhD and a career in science, so I am also a minority in terms of my nationality. I must praise, however, the culture and work ethics in the UK. The British academic system has always been very good to me and in it I have found a home. I have always been treated with respect, given opportunities, given support by some championing mentors and colleagues, and have had the freedom to pursue the science I loved. I am aware that opportunities for women in other countries may not be straight forward, and I hope that collectively we can work towards a more inclusive academia all around the world.

How can publishers, editors, funders and conference organizers better support women scientists?

I wish to see publishers, editors, funders and conference organisers heavily scrutinising their data on gender, actively publishing it, and actively adopting measures to improve it. I would like a gender equality index to be developed by the community and for publishers and funders to report their index regularly. I would like to see employers giving more value to excellent work published in high impact and high equality index journals. I would like funders to provide more data on success rate by gender, to simplify the funding process and to diversify the type of projects (and PIs) they invest in. The question we need to ask is ‘how can we make the system fairer so that the best science is funded and published independently of who you are and who you know?’.

Do you have any advice you would like to share with women starting out in chemical research?

Identify the chemistry you love, be realistic, and find good mentors and collaborators.

- **Identify the chemistry you love**. This is not necessarily obvious. When we do research, we learn a lot about a narrow field. Getting the field right is critical, you need to love what you do! Make the most of internships, summer placements and talk to researchers around you. Only embark on a PhD project when you really know it is something you are very passionate about, and do so in a group with a supportive principal investigator.
- **Be realistic**. Be realistic about what makes you happy, about your priorities in life, about how those priorities align with a career in chemistry research, and about your own strengths and weaknesses. Ask the questions as you go through the different career stages, and be constant and patient in pursuing long term goals. Acknowledge that luck plays a role in any career path, but that being persistent can be more important. Do not give up! We all have had many rejections and often just one chance.
- **Find good mentors and collaborators**. Recognize from day one that you cannot do it alone. I cannot stress enough the importance of having good mentors and collaborators that guide you through, that are honest and critical with you, that teach you along the way and that make the whole process a lot more fun. Reach out to people you like, ask them for advice, ask them to be your mentor, ask them to work together! Take the lead.

Where do you hope to see women in chemistry in 20 years?

I do wish that we can finally tackle the leaky pipeline and that we see a much better gender balance in the chemical sciences at all levels. The data collected so far in gender and chemistry shows that, although the trends are going in the right direction, chemistry in academia is disappointingly bad at retaining and compensating women appropriately and it may well take at least another century to start seeing better numbers. We need adjustments to support tenure of women at a point in their lives when they may also be having and raising children; we need to achieve a better work-life balance in academia; we need to reform the peer review process and make it more open and less biased; we need to make the funding of science more agile and simpler; and we need to revisit the rules of academic appointment and promotion to recognize the huge benefit that diversity brings to science.

*This interview was conducted by the editors of Communications Chemistry*.

